# Local resection of rectal neuroendocrine tumor with first clinical manifestation of giant liver metastasis by transanal endoscopic microsurgery: A case report

**DOI:** 10.1097/MD.0000000000009153

**Published:** 2017-12-15

**Authors:** Gang Xu, Peipei Wang, Yao Xiao, Xin Wu, Guole Lin

**Affiliations:** a Department of Liver Surgery; b Department of General Surgery, Peking Union Medical College (PUMC) Hospital, PUMC and Chinese Academy of Medical Sciences, Beijing, China.

**Keywords:** colorectal, gastrointestinal surgery, liver metastasis, minimally invasive surgery, rectal neuroendocrine tumor, transanal endoscopic microsurgery

## Abstract

**Rationale:**

Rectal neuroendocrine tumor (NET) is a relatively rare tumor. Well-differentiated NETs (G1 and G2) rarely display distant metastasis at initial diagnosis. Currently, treatment for the primary lesions of rectal NETs with liver metastasis remains controversial. The liver metastasis was resected in local hospital. Transanal endoscopic microsurgery (TEM) has emerged as an effective minimally invasive surgery for local resection of lower rectal lesions. Herein, we reported the initial application of TEM to remove the rectal primary lesion in patients with low rectal NETs (G2) with giant liver metastases.

**Patient concerns:**

The patient, a 45-year-old woman, was primarily diagnosed with hepatocellular carcinoma and underwent curative resection of a giant liver lesion in a local hospital. Nevertheless, the postoperative pathologic examination revealed that the lesion was an NET (G2). The colonoscopy then showed a nodule 1.4 cm in diameter, 4 cm above the anal verge, located on the anterior wall of the rectum. The biopsy revealed that the nodule was also an NET (G2). However, the patient did not consent to abdominoperineal resection based on concerns for quality of life.

**Diagnoses:**

Rectal NET with liver metastasis.

**Interventions:**

The patient underwent curative resection of liver metastasis. And, TEM was adopted to resect the primary tumor in rectum.

**Outcomes:**

The patient has been disease-free for 2 years with a good quality of life and presents no local recurrence in the rectum.

**Lessons:**

TEM is an appropriate palliative operation for therapy of rectal NETs with distant metastases, especially for primary rectal NETs located in low rectal.

## 1 Introduction

Neuroendocrine tumors (NETs), formerly called carcinoid tumors, are located most commonly in the gastrointestinal tract and bronchopulmonary system.^[1]^ Rectal NETs account for 13.7% of all NETs.^[2]^ Due to the widespread use of colonoscopy as a screening tool, the rate of detection of colorectal NETs is increasing.^[3,4]^ Basing on the Ki-67 proliferation index or the mitotic count, in 2010, the tumors were classified as NET G1, NET G2, and poorly differentiated neuroendocrine carcinoma (NEC G3) by the World Health Organization.^[5]^ Rectal NETs are mostly asymptomatic and are commonly diagnosed during routine health examinations.^[6,7]^ Most of rectal NETs (80–88%) are localized, whereas the remaining NETs (12–20%) are diagnosed with regional lymph node spread and/or distant metastases.^[8]^ According to the SEER database analysis, the median survival of distant metastatic disease was 33 months in patients with NET G1-G2 but was only 5 months in patients with NEC G3.^[9]^ Oncological lymph node dissection and/or debulking surgery or locoregional therapies of primary tumor resection have been associated with a better prognosis.^[10,11]^ Herein, we report a case of rectal NET (G2) with first clinical manifestation of giant liver metastasis that was treated with a curative resection of liver metastasis and local resection of the primary rectal tumor by transanal endoscopic microsurgery (TEM) with long-term follow-up.

## 2 Case report

**Figure 1:**
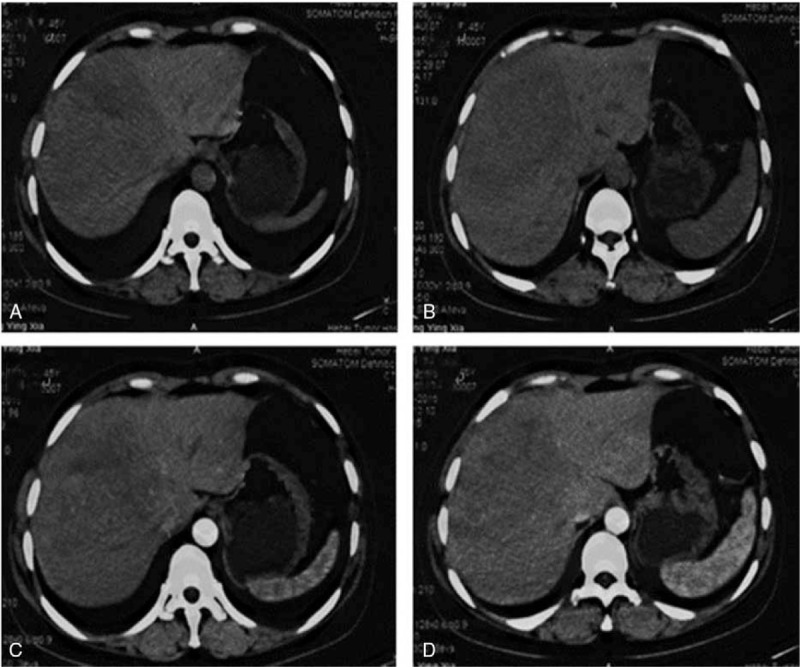
CT: (A, B) Plain phase. A tumor approximately 11.0 cm in diameter was found in the right lobe of the liver; (C, D) Arterial phase. The peri-tumor area was slightly enhanced.

**Figure 2:**
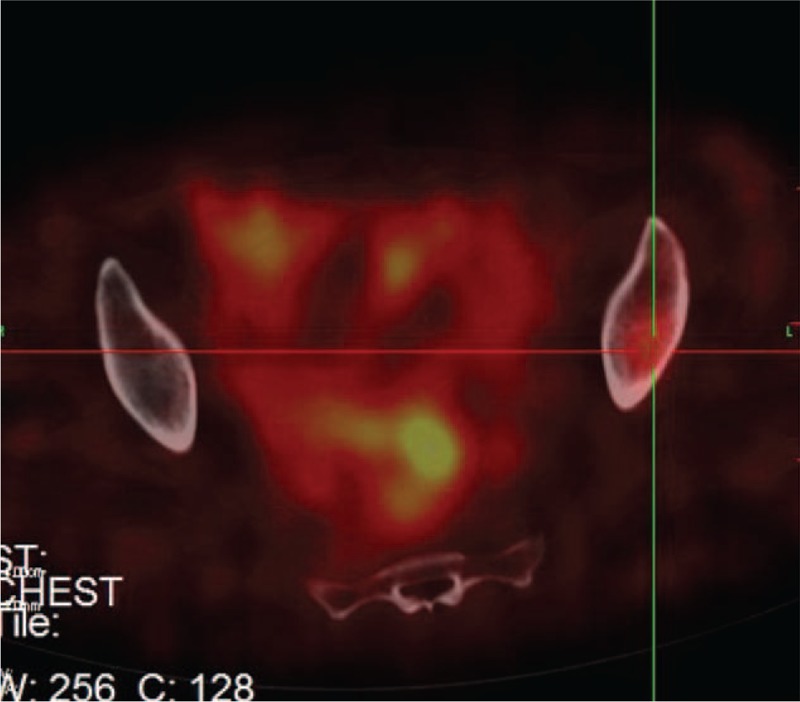
SRS: Abnormal uptake in the left ilium.

In April 2015, a 45-year-old Chinese female presented with intermittent constipation and hematochezia and was admitted to our institution. Three months before the admission, the patient was seen in the local hospital with a chief complaint of progressive abdominal distension for the past 3 months along with a loss of appetite. Computed tomography (CT) of the liver (Fig. 1) showed a tumor approximately 11.0 cm in diameter in the right lobe of the liver. The results for blood routines and liver function and coagulation tests were within normal ranges. Tumor marker determination was also normal. Doctors suspected that the liver tumor was a primary hepatic tumor, such as hepatocellular carcinoma. The patient underwent partial hepatectomy in the local hospital. However, the postoperative pathology revealed that the hepatic lesion was a moderately differentiated NET (G2). Immunohistochemistry findings included HepPar-1 (−), AFP (−), CK7 (−), CK20 (−), CK (+), CD56 (+), Ki-67 (7%), Syn (+), CgA (−/+). A subsequent colonoscopy discovered a nodule 1.4 cm in diameter, located on the anterior wall of the rectum, 4 cm above the anal verge, and histological examination confirmed the diagnosis of rectal NET (G2). These findings indicated that the liver tumor might have metastasized from the asymptomatic rectal NET. Magnetic resonance imaging was also performed for the evaluation of the rectal tumor and revealed a small mucosal tumor at the rectum; no lymph nodes were found in the pelvic cavity. Therefore, the rectal NET was staged T1N0 (data not shown). The patient attended our clinic for further treatment. Somatostatin receptor scintigraphy (SRS) showed abnormal uptake in the left ilium (Fig. 2), which was considered not to be a metastasis, as indicated by multidisciplinary treatment consultations. As the tumor was located low in the rectum, 4 cm above the anal verge, abdominoperineal resection should be performed. When the patient does not consent to a radical procedure based on concerns for quality of life, TEM becomes an optimal solution.

**Figure 3:**
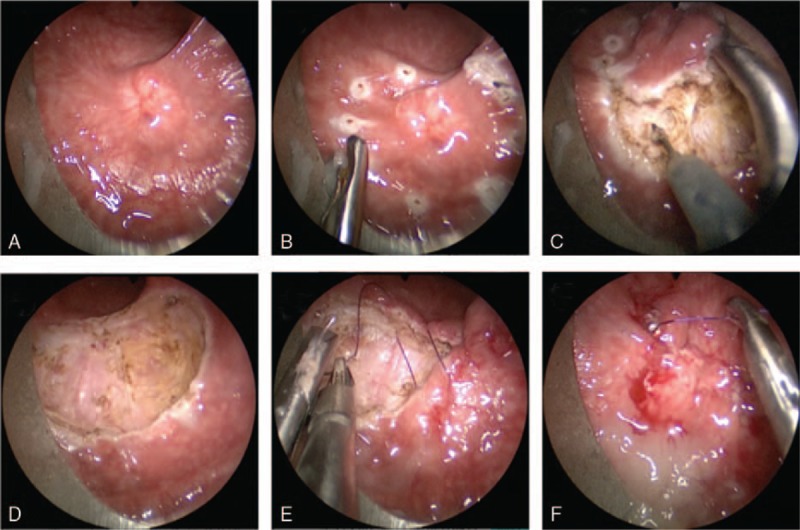
Details of the tumor resection by transanal endoscopic microsurgery. (A) Rectoscopic view of the tumor; (B) A 1 cm resection margin was marked around the lesion before excision by needle diathermy; (C, D), Full-thickness excision was performed using an ultrasonic dissector; (E, F), Defects in the rectal wall were closed using running sutures of 3/0 absorbable monofilaments.

**Figure 4:**
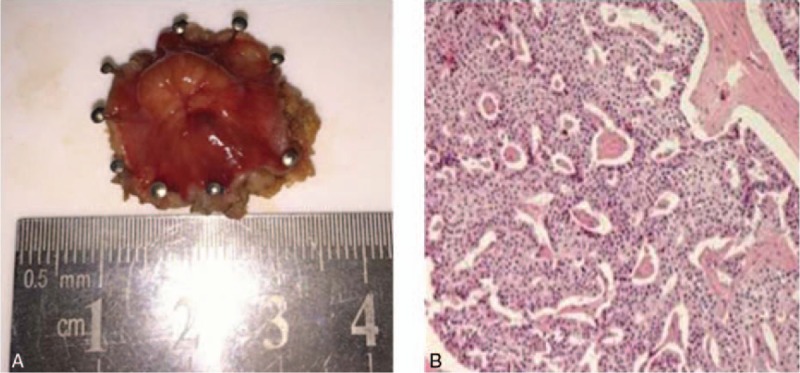
Surgical specimen and bioptic microscopic images. (A) Surgical specimen of the rectal NET measured proximately 14 mm × 10 mm × 5 mm. The tumor exhibited a heterogeneous shape and surface; (B) Pathological image revealing neuroendocrine tumor cells beyond the muscularis propria and invading adipose tissue (hematoxylin and eosin, ×100).

Rigid sigmoidoscopy was used to confirm the location of the nodule, followed by TEM under general anesthesia. The patient was placed in the prone position with the lesion placed at the bottom of the operating field. The surgery was performed as previously described by Buess et al,^[12]^ using the Buess original TEM system (Richard Wolf GmbH, Knittlingen, Germany). After marking the resection area with coagulation dots using a needle cautery, ensuring a free margin area of 1 cm, the tumor was removed one at a time with a full-thickness excision. Defects in the rectal wall were irrigated and closed using running sutures with 3/0 absorbable monofilaments (Fig. 3). The surgery was completed within 45 minutes with an approximate blood loss of 10 mL. After surgery, the resected specimen was pinned out on a cork board (Fig. 4). No analgesic was required postoperatively. An elementary diet was initiated on the second day, and the patient was dismissed from PUMCH (Peking Union Medical College Hospital) 2 days after the surgery in good clinical condition.

**Figure 5:**
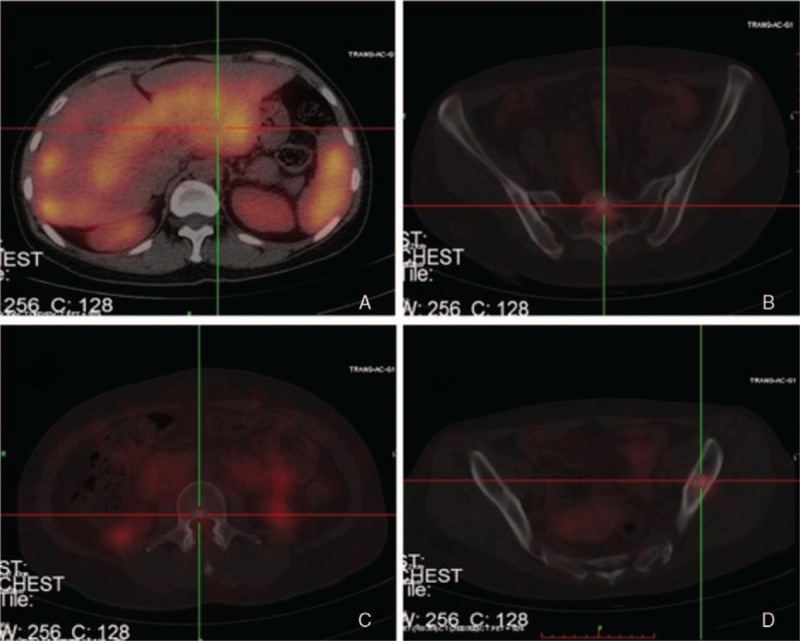
Somatostatin receptor scintigraphy. (A) Abnormal uptake in the liver; (B, C) Abnormal uptake in the lumbar spine; (D) Abnormal uptake in the left ilium.

The postoperative pathology revealed that the tumor, 12 mm in diameter, was located beyond the muscularis propria and had invaded adipose tissue with lymphovascular invasion (Fig. 4). However, the deep and lateral surgical margins were completely tumor-free. Tumor cells showed a moderate cell proliferation index (Ki-67 8%). Immunohistochemistry studies included the following: AE1/AE3 (+), PGP9.5 (+), Syn (+), CK20 (−), CD56 (+), CgA (+), CD34 (−). The final diagnosis was rectal NET G2 (carcinoid). The patient was informed to a radical abdominoperineal resection, but she did not consent to this treatment planning. The patient was followed up regularly as an outpatient with evaluation by CT and endoscopic examination every 3 months. Two years later, the patient presented with back pain. And the SRS showed pathologic uptake in the liver, lumbar spine, and left ilium (Fig. 5). Nevertheless, no local recurrence in the rectum was found using digital rectal examination.

## 3 Discussion

Rectal NET is a rare tumor with an incidence rate of 0.14 to 0.76/100,000 cases.^[7,13]^ Their natural history is characterized by lack of symptoms from the primary tumor; thus, NETs are notoriously difficult to diagnose. Approximately 12% to 20% of rectal NETs are diagnosed with regional lymph node spread and/or distant metastases (21% G1, 30% G2, and 50% NEC).^[8]^ The 5-year survival in distant metastatic disease is 35% in well-differentiated to moderately differentiated NET, and the median survival is 33 months.^[9]^ Liver surgery is generally proposed with curative intent to all patients with operable well-differentiated (NET G1/G2) metastases from NET regardless of the site of origin (e.g., foregut, midgut, hindgut).^[14]^ Adjuvant therapies such as chemotherapy are sufficient to confirm metastatic rectal NET cases without obstruction or rectal hemorrhage, as suggested by the ENETS (European Neuroendocrine Tumor Society) guidelines.^[15]^ Smith et al^[16]^ supported this idea, and their research findings suggest that removing high-grade rectal NET lesions does not improve the prognosis of the patients. Meanwhile, the NCCN (National Comprehensive Cancer Network) proposed that certain managements be determined based on the resectability and symptoms of these patients. Primary and metastatic lesions should undergo radical resection whenever possible, while palliative resection is conducted only when resective problems are presented, such as combinations of severe local symptoms.^[17]^ Durante et al^[11]^ reported that early primary tumor resections, including oncological lymph node dissection and/or debulking surgery or locoregional therapies, were associated with a better prognosis. In our case, the patient underwent partial hepatectomy with pathology confirmation of a hepatic NET G2 tumor. Meanwhile, the colonoscopy showed a nodule 1.4 cm in diameter, located on the anterior wall of the rectum, 4 cm above the anal verge. The biopsy showed the nodule was also an NET (G2). SRS revealed abnormal uptake only in the left ilium. According to the results of the patient's examinations, we considered that the giant liver tumor might have metastasized from the asymptomatic rectal NET. However, well-differentiated NETs (G1 and G2) rarely display distant metastasis. Tumor size, depth of invasion, and lymph node involvement significantly predict malignant behavior in localized rectal NETs.^[18]^ According to 1 analysis of the literature, metastases were observed in 10% to 15% of patients with rectal NETs measuring 1.0 to 2.0 cm.^[19]^ Another article reported that metastases occurred in only 2% of tumors smaller than 2 cm that had not invaded the muscularis propria, compared with 48% in tumors invading the muscularis layer. Many previous studies have reported the presence of lymphovascular invasion in rectal NETs as a strong risk factor for metastasis.^[20–22]^ Magnetic resonance imaging revealed that the rectal NET was staged as T1N0, and biopsy information through colonoscopy was limited. To confirm the primary tumor and control of local complications (e.g., hematochezia) and to avoid local complications, such as obstruction, TEM was performed to remove the rectal lesion completely. Another case report described a patient with rectal NET graded as G1 discovered by sporadic metastasis in the liver. The patient showed good progress with no recurrence after undergoing hepatectomy and endoscopic resection of the rectal NET.^[23]^ Nevertheless, the intrinsic limitations of the conventional endoscopic polypectomy result in a high chance of incomplete resection.^[24]^ Since its introduction by Buess et al^[25]^ in 1983, TEM has emerged as an effective minimally invasive surgery for local resection of rectal lesions. This technique enables full-thickness excision and ensures accurate resection with sufficient margins by applying the delicate instruments under superior visualization. In addition, TEM allows suturing of rectal wall defects after tumor resection, thus securing sufficient excision without worrying about rectal wall perforation. In comparison with endoscopic resection methods, including advanced techniques of endoscopic mucosal resection with cap and endoscopic submucosal resection with band ligation, TEM enables a much larger extent of resection, ensuring more satisfactory oncological results for lesions with malignant potential. In our case, the local lesion was resected completely. The postoperative pathology revealed a tumor 12 mm in diameter that was located beyond the muscularis propria and had invaded the adipose tissue with lymphovascular invasion. According to the previous research, invasion of the muscularis propria and lymphovascular system are risk factors for distant metastasis. We then considered that the liver tumor might have metastasized from the asymptomatic rectal NET. The invasion may also be the reason why a giant liver metastasis was found in the liver despite the primary rectal tumor being well-differentiated and small in size. Systemic chemotherapy has not shown efficacy against metastatic gastrointestinal NETs^[26,27]^; an analysis showed long progression-free survival of patients who received everolimus plus octreotide, but further verification of these results is required.^[28]^ Adjuvant chemotherapy is only recommended in the treatment of patients diagnosed with NECs and NETs G2 with Ki-67>15%.^[28]^ The patient did not consent to a radical rectal resection. On the basis of these facts, our patient has been followed up carefully without chemotherapy.

To our knowledge, this is the first reported case of rectal NET with liver metastasis being managed by TEM. In our case, a high standard local tumor resection of the rectal NET was performed efficiently, with minimal operative trauma. The rapid and smooth recovery of the patient also indicated the advantages of the TEM technique. In this case, in addition to controlling the local complications, TEM, with its full-thickness excision and partial mesorectal excision, also provides more pathologic information, aiding in diagnosis and prognosis evaluations. The patient has been disease-free for 2 years with a good quality of life and presents no local recurrence in the rectum, which may imply that TEM is an appropriate palliative operation for therapy of rectal NETs with distant metastases, especially for primary rectal NETs located in low rectal. However, for further validation of the effects of this new strategy, prospective studies are needed in the future.

## 4 Conclusion

Treatment for the primary lesions of rectal NETs with liver metastasis remains controversial. As the unsatisfactory therapeutic effects and limited ranges of clinical applications of nonsurgical treatments, surgery remains the major current option for primary rectal NETs. The patient was always informed to a radical abdominoperineal resection when the rectal NETs located in lower rectum. Considering the surgical trauma and anal function, some patients say no to radical surgical treatment. In this case, TEM in combinations with careful followed-up achieve satisfactory anal and urinal function and good long-term prognosis. For the lower rectal NETs with distant metastasis, TEM could be the initial attempt as a debulking surgical treatment and maintain satisfactory anal and urinal function-preserving. Moreover, long-term outcomes still need to be determined using a larger series of patients. With further intensive study of targeted therapy and high-quality combination therapy, it is possible that the prognosis and quality of life of rectal NETs patients in advanced stages or with metastasis may be improved in the future.
